# Endometriosis Cell Spheroids Undergo Mesothelial Clearance in a Similar Manner to Ovarian Cancer Cell Spheroids

**DOI:** 10.3390/cells14100742

**Published:** 2025-05-19

**Authors:** Allison A. Kloeckner, Sarah R. Walker

**Affiliations:** Department of Molecular, Cellular and Biomedical Sciences, University of New Hampshire, Durham, NH 03824, USA

**Keywords:** endometriosis, mesothelial clearance, epithelial cells, in vitro model

## Abstract

Endometriosis is a gynecological disease characterized by the presence of endometrium-like cells located outside the uterus. The most widely accepted theory for endometriosis development, retrograde menstruation, does not account for extra-pelvic lesions or ones found on other organs in the peritoneal cavity. Similar to ovarian cancer, endometriosis cells can interact with the mesothelial cells of the peritoneal cavity. In ovarian cancer metastasis, ovarian cancer cell spheroids attach and push away the mesothelial cells lining the peritoneal cavity, clearing the mesothelial layer. Since endometriosis cells are known to interact with the mesothelium, we hypothesized that endometriosis cells would be able to form spheroids capable of undergoing mesothelial clearance. To test this, we designed an in vitro mesothelial clearance assay using endometriosis spheroids and a mesothelial cell monolayer. Our results demonstrate that normal and endometriotic epithelial cell spheroids can perform mesothelial clearance similar to ovarian cancer spheroids, though normal endometrial cells do not clear as well as endometriosis cells. Additionally, we demonstrated that our mesothelial clearance assay can test potential pharmacological therapies for endometriosis prior to clinical trials. These results give insight into the development of endometriosis lesions, but further research is needed to determine the mechanisms behind mesothelial clearance in endometriosis.

## 1. Introduction

Endometriosis is a gynecological disease that affects around 190 million women and people assigned female at birth during their reproductive years [1]. This disease is defined by the presence of endometrial-like stroma and epithelial cells in any extra-uterine location [1,2]. The endometriotic lesions that form result in a chronic, inflammatory condition with many patients experiencing debilitating pain [1,2,3]. Common symptoms include pelvic pain, infertility, and many others that negatively affect physical, mental, and social well-being [3,4]. While most lesions are found within the abdomen, lesions can be found in parts of the gastrointestinal system and outside the abdominal area as well [1]. The varying locations of endometriotic lesions present an interesting perspective through which to research endometriosis. Determining how extra-abdominal lesions form could shed light on the pathogenesis of the disease.

As the endometrium, the lining of the uterus, is a very complex tissue, understanding its function in the body is necessary to fully comprehend how it is involved in the development of endometriosis [5,6,7]. The main role of the endometrium is to prepare itself for embryo implantation and, if implantation is successful, to maintain the pregnancy [6]. Since endometrium plays an important part in sexual reproduction, it is heavily influenced by ovarian steroid hormones [6]. Estrogen and progesterone prompt modifications of the endometrium, causing it to thicken or thin depending on the menstrual phase [5,6]. When pregnancy does not occur, the change in steroid hormones causes the endometrium to shed through menstruation [5,6]. Sampson’s theory on how endometriosis occurs suggests retrograde menstruation is responsible [8,9]. Instead of flowing out through the vagina, during retrograde menstruation, shed endometrium cells travel back up the uterus and into the fallopian tubes, implanting in the peritoneal cavity, thus leading to the formation of endometriotic lesions [7,8,10]. However, retrograde menstruation is estimated to occur in almost 90% of women, and therefore, it cannot fully explain all instances of endometriosis and the varying locations where endometriotic lesions have been found [7,10,11]. This led the way for alternative theories on the pathogenesis of endometriosis, yet none fully encapsulates all instances of endometriosis and the interaction between the lesions and the peritoneum [12]. Specifically, the attachment of endometriosis lesions to the mesothelial layer of the peritoneal cavity left many questions unanswered. How the lesions were able to attach to the mesothelial layer to initiate implantation was not well understood.

In order to investigate the interaction between the monolayer of mesothelial cells that line the peritoneal cavity, an adequate in vitro model of endometriotic lesions that can represent the complexities of the disease is essential [13]. Although benign, endometriotic lesions share similarities with tumors, such as increased cell proliferation, neoangiogenesis, and reduced apoptosis [10,14]. Notably, endometriosis has been associated with ovarian cancer, the deadliest gynecological malignancy for women [15,16]. These similarities between endometriotic lesions and ovarian tumors warrant the application of ovarian cancer research approaches for the study of endometriosis.

Previous studies have highlighted the benefits of utilizing three-dimensional (3D) models of cancer to better represent what is occurring in vivo [17,18]. While two-dimensional (2D) models have been a vital part of furthering biological research, they often have differences in gene expression and drug response due to their inability to represent the complex microenvironment that surrounds tumors in vivo [17]. Recently, 3D cell culture methods have become more popular due to the need for a complex in vitro system that accurately represents cell–cell interactions [13,18]. Three-dimensional spheroids are clusters of cells that can comprise either exclusively tumor cells or a combination of tumor, stromal, and immune cells [13,18]. These spheroids self-assemble into circular aggregates that do not attach to any plate surfaces [13]. A review of publications in which 3D spheroid models of ovarian cancer were used showed that the most common spheroid-formation method was to culture ovarian cancer cells in pre-coated ultra-low-attachment (ULA) plates [17,19]. These ULA-plate-generated spheroids showed similarities to the spheroids that are typically found in the ascites of ovarian cancer patients [18].

The concept for 3D spheroid models does not only apply to ovarian cancer. In 2014, researchers established in vitro 3D spheroid models of endometriosis using a well-characterized endometriosis epithelial cell line, 12Z, and an ovarian endometriosis epithelial cell line, EEC16, isolated from a patient with severe endometriosis [20]. Spheroids from this study were made in non-adherent conditions using polyHEMA-coated cell culture plastics [20]. The spheroids were compared to 2D monolayer cell cultures and were found to have differential gene expression of endometriosis-related genes [20]. Furthermore, the molecular and histological features of these spheroids were more similar to human endometriosis lesions than the 2D monolayer cultures [20]. In addition, Song et al. demonstrated the formation of endometriosis spheroids comprising 12Z epithelial cells and either uterine stromal cells or endometriotic stromal cells [13]. In both combination spheroids, the stromal cells aggregated in the center of the spheroid, with the 12Z cells surrounding them on the outside [13].

The establishment of 3D spheroid models has been integral in enhancing endometriosis research, but a need still exists for 3D models that enable researchers to study the interactions between the microenvironment and these 3D spheroids [13]. Specifically, models that can show the interactions between endometriosis cells and the peritoneal mesothelial cells are needed to better understand the generation of peritoneal endometriotic lesions. The mesothelium is a monolayer of cells that acts as a barrier around all of the organs in the peritoneal cavity [21]. It was initially believed that endometriotic lesion attachment to the peritoneal mesothelium was only able to occur when the mesothelial layer was damaged [9]. However, Witz et al. demonstrated endometrial stromal cells could attach to undamaged mesothelium [9]. They mechanically dispersed endometrial stromal and epithelial cells onto an intact mesothelium of peritoneal explants and saw adhesion to the mesothelium occurred only with stromal cells [9]. To build on this, Witz et al. have also shown endometrial stromal cell adherence to the peritoneal mesothelium and provided evidence to support that they are capable of invading past the mesothelium [22]. While these studies did not use endometriosis cells, they provided data to support the possibility that the formation of endometriotic lesions on the peritoneal mesothelial layer could occur.

In the study by Song et al. mentioned previously, the invasion capability of the combination endometriosis spheroids was also assessed [13]. In their 3D invasion model, the spheroids invaded a Matrigel matrix and penetrated a layer of mesothelial cells [13]. Interestingly, the endometriotic stromal cells were consistently located at the leading edge of invasion and were the first cells to breach the mesothelial layer, dragging the 12Z cells behind them [13]. These data concurred with the data from non-endometriotic mesothelial invasion tests in which the stromal cells were the driving force of attachment and invasion.

Our current research aims to study the ability of endometriotic epithelial cells themselves to disrupt mesothelial cell monolayers. Our methods do not investigate the invasive properties once the mesothelial layer is breached, which has previously been shown. We believe that epithelial endometriotic cells can disrupt or clear the mesothelial cell layer, similar to how ovarian cancer metastasis occurs. Mesothelial cells act as a barrier for endometriotic lesion formation; thus, the endometriotic cells must interact with this monolayer for lesion implantation. The ability of ovarian cancer spheroids to clear the mesothelial layer of cells in the peritoneal cavity gives them access to the underlying extracellular matrix, which they must breach if the cancer is to spread to locations outside the peritoneal cavity. Showing that endometriosis spheroids can clear a mesothelial monolayer offers a potential explanation for the mechanisms that lead to extra-pelvic endometriotic lesions.

To study this, we have developed a straightforward in vitro mesothelial clearance assay that eliminates the need for scaffolds and stable fluorescently labeled cells, making it a low-cost and less time-consuming method. An immortalized endometriosis cell line, 12Z, along with an immortalized endometrium cell line, hEM3, were used to generate 3D spheroids. Spheroids were also made using the ovarian cancer cell line OVCAR8 as a positive control for mesothelial clearance. Our clearance method permits both the study of genes that promote mesothelial clearance and the effectiveness of drugs that inhibit mesothelial clearance.

## 2. Materials and Methods

### 2.1. Cell Lines

Two endometrial epithelial cell lines, 12Z (Applied Biological Materials, Ferndale, WA, USA) and hEM3 (kindly provided by Dr. Ie-Ming Shih [23]), were used. 12Z cells are an immortalized endometriosis cell line, and hEM3 cells are an immortalized normal endometrium cell line. 12Z cells were grown in DMEM High-Glucose media and supplemented with 10% FBS and 1x Antibiotic–Antimycotic. hEM3 cells were grown in RPMI 1640 media and supplemented with 15% FBS, 1x Antibiotic–Antimycotic, and 1x MEM Non-Essential Amino Acids Solution. LP9 Mesothelial cells (a kind gift from Martin Sattler, Dana-Farber Cancer Institute, Boston, MA USA) were used to represent the monolayer of mesothelial cells that line the peritoneal cavity in our in vitro assay. LP9 cells were grown in media containing a 1:1 ratio of Human Fibroblast Expansion Media (Gibco, Grand Island, NY, USA) and Media 199, supplemented with 15% Iron-Enriched Calf Serum, 1x Antibiotic–Antimycotic, 4 μg/mL Hydrocortisone, and 10 ng/mL Human EGF (Peprotech, Cranbury, NJ, USA). The ovarian cancer cell line OVCAR8 (kindly provided by Ronnie Drapkin, University of Pennsylvania) was used as a comparison for the activity of the endometriosis cell lines. OVCAR8 cells were cultured in RPMI 1640 media that was supplemented with 10% FBS and 1x Antibiotic–Antimycotic. Ishikawa cells (Millipore Sigma, Burlington, MA, USA) were grown in MEM media and supplemented with 5% FBS, 1x Antibiotic–Antimycotic, 1x MEM Non-Essential Amino Acid Solution, and 2 mM L-Glutamine. ZR-75 (kindly provided by Kornelia Polyak, Dana-Farber Cancer Institute) and mouse embryonic fibroblasts (*fl/fl STAT3*) (kindly provided by David Frank, Emory University) were grown in DMEM with 10% FBS and 1x Antibiotic–Antimycotic. All cells were grown in a 37 °C environment supplied with 5% CO_2_ and tested for mycoplasma contamination.

### 2.2. Drug Treatment

Atorvastatin (Selleckchem, Houston, TX, USA), simvastatin (MedChemExpress, Monmouth Junction, NJ, USA), and lovastatin (MedChemExpress, Monmouth Junction, NJ, USA) were dissolved in dimethyl sulfoxide (DMSO) to stock concentrations of 10 mM. All drugs were stored at –20 °C. For the mesothelial clearance assay, spheroids were treated with either 10 µM Atorvastatin, 10 µM Simvastatin, or 10 µM Lovastatin and incubated at 37 °C for 24 h before spheroids were transferred on top of LP9 cells.

### 2.3. 3D Spheroid Culture

12Z, hEM3, and OVCAR8 spheroids were generated by plating 1 × 10^4^ cells in 100 µL media in U-bottom ULA plates (Greiner Bio-One, Pittsburgh, PA, USA) for 72 h unless otherwise indicated.

### 2.4. Cell Viability Staining

NucBlue and propidium iodide (PI) cell stains (ReadyProbes^TM^ Cell Viability Imaging Kit, ThermoFisher Scientific, Waltham, MA USA) were used to visualize the viability of cells that made up the spheroids. NucBlue stains the nucleus of all cells, dead or alive, causing them to fluoresce blue. To visualize these stained cells, images were taken on an EVOS FL Auto 2 using the DAPI channel. PI is a red cell stain that was used to differentiate the dead cells from the live cells, as it stains only the nuclei of dead cells that have damaged membranes. The RFP channel on the EVOS was used to capture images of the cells stained with PI.

### 2.5. Mesothelial Clearance Assay and Analysis

**Figure 1:**
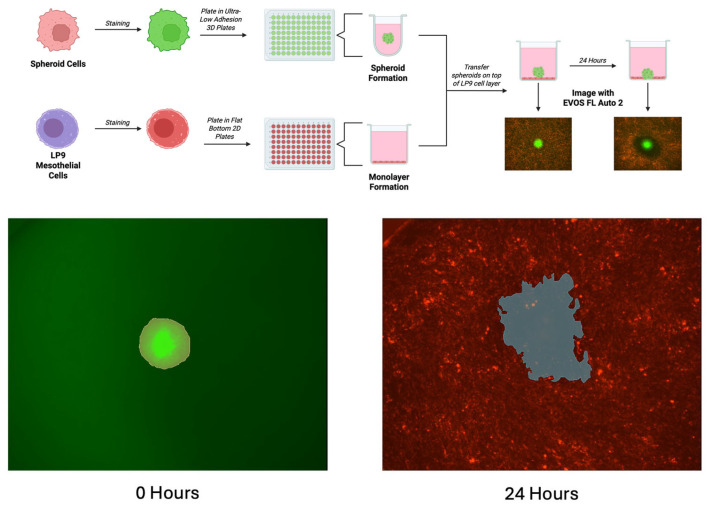
Mesothelial Clearance Assay and Analysis Methods. Top: Schematic of mesothelial clearance. Endometriosis cells are stained green and plated in U-bottom ULA plates to form spheroids. LP9 mesothelial cells are stained red and plated in flat-bottom tissue-culture-treated plates to form a monolayer. After 72 h, the media is removed from the LP9 monolayers, and spheroids are transferred on top. After a 30 min incubation to promote spheroid attachment, images are taken using the EVOS microscope at the initial timepoint and again 24 h later. Bottom: 3D Slicer software (version 5.2.2) is used to measure the area of the spheroid at the initial image timepoint (left, yellow line) and the clearance area at the 24 h timepoint (right, blue line) to find the clearance ratio of cleared area/spheroid area.

A schematic of this assay can be seen in Figure 1. A total of 4 × 10^6^ LP9 cells were resuspended in 4 mL of media before being stained red with the Invitrogen Vybrant^TM^ Dil cell-labeling stain (Waltham, MA, USA). A total of 3 × 10^6^ OVCAR8 or endometrial cells were resuspended in 3 mL of their respective media and stained green with the Invitrogen Vybrant^TM^ DiO cell-labeling stain (Waltham, MA, USA). All cells were then incubated at 37° for 20 min. Cells were centrifuged and washed with PBS twice. After washing, the PBS was removed, and LP9s were resuspended in 15 mL media while the spheroid cells were resuspended in 3 mL of their respective media. LP9 cells were plated at a volume of 100 µL per well in a clear, flat-bottom, tissue-culture-treated 96-well plate for 72 h. For spheroid plating, 1 mL of the stained cell solution was combined with 9 mL of media and plated at a volume of 100 µL or 50 µL if adding drugs, as described above. The cells were incubated for 48–72 h until spheroids formed. After incubation, the media was removed from the LP9 mesothelial cells, and the spheroids, along with their media, were transferred on top of the mesothelial monolayer using an Integra VIAFLO electronic pipette with the Integra Wide-Bore pipette tips (Hudson, NH, USA). The cells were incubated for 30 min to allow for spheroid adhesion to the monolayer. The Invitrogen EVOS FL Auto 2 microscope (Waltham, MA, USA) was used to image the wells using the GFP and RFP channels and the 4× objective at 0 and 24 h by creating a scan protocol. To measure the size of the spheroids and the area cleared by them, the 3DSlicer image computing platform was used (www.slicer.org, accessed on 22 February 2023, version 5.2.2). The Segment Editor was used to trace the outlines of the spheroids and cleared area, while the Segment Statistics were used to quantify the measured areas. The initial spheroid size at 0 h and the clearance area at 24 h were measured in mm^2^. The clearance ratio was calculated as the area cleared at 24 h divided by the area of the spheroid at 0 h. Each experiment represents 5–8 spheroids per condition per experiment replicate (per N), as indicated in the figure legends. Prism by GraphPad (version 10.4.2) was used to run one-way ANOVA analyses on the clearance ratios (*p* < 0.05), followed by Dunnett’s multiple comparison tests. Once analyzed, ImageJ (version 1.54g) was used to enhance clearance images for visualization purposes only. The brightness and contrast of the images were increased, and the color balance values were adjusted to augment the different cell stains. Unenhanced images can be found at FigShare (https://figshare.com/articles/figure/Complete_Image_Sets/28796033).

## 3. Results

### 3.1. 12Z Endometriosis and hEM3 Endometrial Cells Form Spheroids in 3D Cell Culture

**Figure 2:**
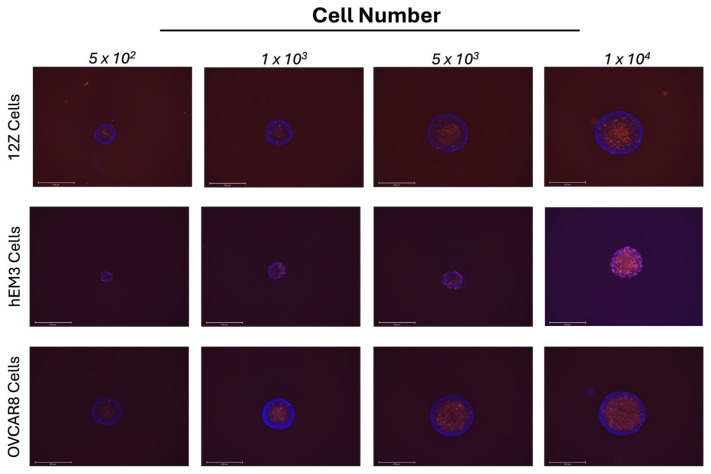
Increasing cell number increases the proportion of dead cells to live cells in spheroids. 12Z, hEM3, and OVCAR8 cells were plated in 3D at varying cell densities per well. On day five, NucBlue (all cells) and PI (dead cells) stains were added, and spheroids were imaged on the EVOS using the 4× objective.

To study endometriosis, we wanted to establish a 3D in vitro model. It is well-accepted that 3D cell cultures better represent the interactions between cells as well as interactions between cells and their environment. OVCAR8 ovarian cancer cells form spheroids when grown in ULA plates and were used as a control to compare against the 12Z and hEM3 cells. To determine if the endometrial 12Z and hEM3 cells would be able to form spheroids in 3D cell culture, cells were plated in 2D and 3D and imaged. Both cell lines were able to form spheroids (Supplemental Figure S1). Next, 12Z and hEM3 cells were plated at varying cell densities in U-bottom ULA plates. The spheroids were then stained with NucBlue and PI to assess their morphology and composition of live versus dead cells (Figure 2). At all cell densities, hEM3 cells form smaller spheroids compared to 12Z and OVCAR8 cells. All spheroids were round with smooth outer borders. These results are consistent with ovarian cancer cell spheroids. PI staining of 12Z and OVCAR8 spheroids demonstrated that the spheroid centers were composed of dead cells (red), while the NucBlue staining in combination with the PI showed live cells (blue) located mainly on the outer portions of the spheroid. However, hEM3 spheroids had more dispersed dead cells (red) that were sometimes found along the outer portions of the spheroid in addition to the center. Increasing cell numbers appeared to increase the proportion of dead cells in comparison to live cells in all cell types.

### 3.2. 12Z and hEM3 Spheroids Can Execute Mesothelial Clearance

Most endometriosis lesions are attached to the pelvic peritoneum. We aimed to expand on past research that showed that only endometrial stromal cells were the driving force of attachment and invasion to mesothelial cell layers. Therefore, we developed an in vitro mesothelial clearance assay to determine if spheroids made up of either 12Z or hEM3 epithelial cells could clear a monolayer of mesothelial cells similar to ovarian cancer spheroids. OVCAR8 ovarian cancer cells were used as a positive control as they are capable of undergoing mesothelial clearance [24].

**Figure 3:**
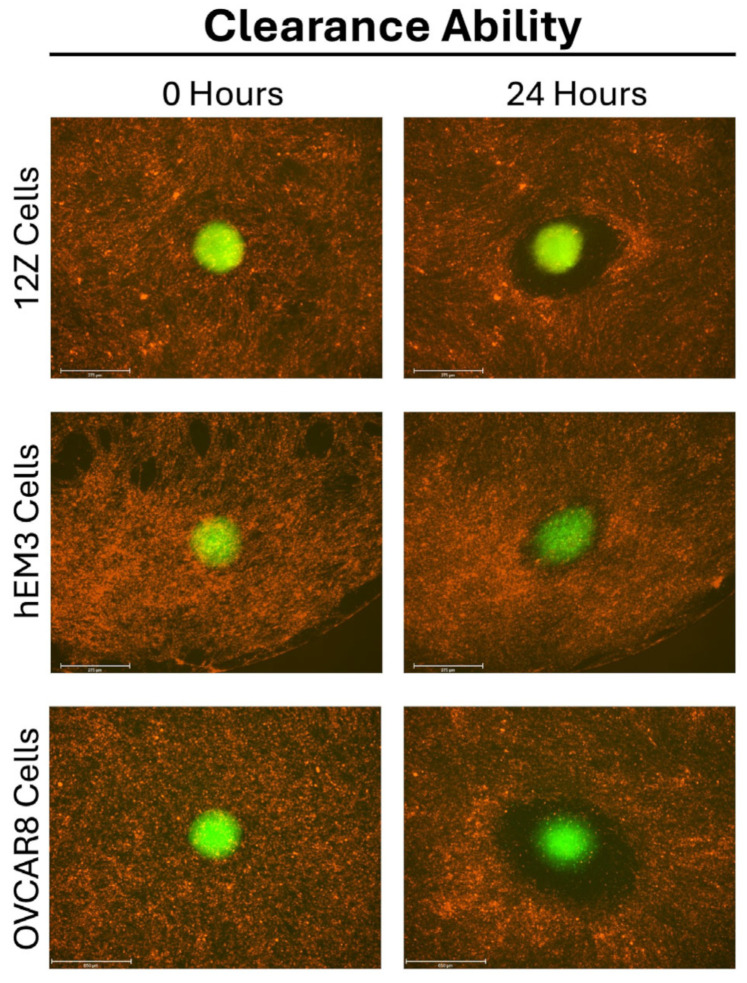
12Z, hEM3, and OVCAR8 spheroids undergo mesothelial clearance. LP9 mesothelial cells were stained red and grown as a monolayer. 12Z, hEM3, and OVCAR8 cells were stained green and plated in 3D to induce spheroid formation. After 72 h, spheroids were transferred on top of the LP9 monolayer, and images were taken at 0 and 24 h using the 4× objective.

We found that both 12Z and hEM3 endometrial epithelial cell spheroids cleared the monolayer of LP9 cells after 24 h (Figure 3). The dark space surrounding the spheroid is the area that the spheroid has cleared by pushing away the mesothelial cells to create a space. We found that after 24 h, the spheroids changed morphology and switched from a tight circular-shaped spheroid to a more oval shape with cells that were not as tightly packed. In some 24 h images, the spheroid cells can be seen dispersed throughout the cleared area. Time-lapse videos of 12Z, hEM3, and OVCAR8 cells suggest that endometriosis cells clear mesothelial cells in a manner similar to ovarian cancer spheroids (Supplemental Figures S2–S4). Interestingly, we also analyzed the ability of Ishikawa endometrial cancer cells to undergo mesothelial clearance, as these cells are often used in endometriosis studies. Unlike the other cells, Ishikawa spheroids are unable to clear the mesothelial cell monolayer beyond the boundary of the sphere (Supplemental Figure S5).

### 3.3. Clearance Ratio Has an Inverse Relationship with Initial Spheroid Size

After determining that endometrial spheroids could perform mesothelial clearance, we wanted to investigate what impact spheroid size had on the clearance ratio. The results of the NucBlue and PI staining (Figure 2) suggest that the proportion of dead cells to live cells increased as the spheroid size increased. Therefore, we plated different spheroid cell densities per well, and the mesothelial clearance assay was then executed.

**Figure 4:**
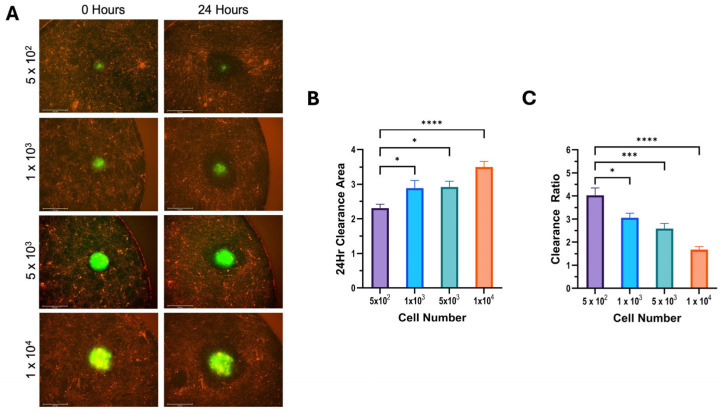
Increasing 12Z cell number increases cleared area but decreases clearance ratio. A mesothelial clearance assay was performed, plating 12Z cells at the indicated spheroid cell numbers. (**A**) Representative images of 0 and 24 h of mesothelial clearance using the 4× objective. (**B**) Clearance area and (**C**) clearance ratio were determined. Data are shown as mean +/− SEM. Data represent a minimum of 5 spheroids per N, N = 3; * *p* ≤ 0.05, *** *p* ≤ 0.001, **** *p* ≤ 0.0001.

**Figure 5:**
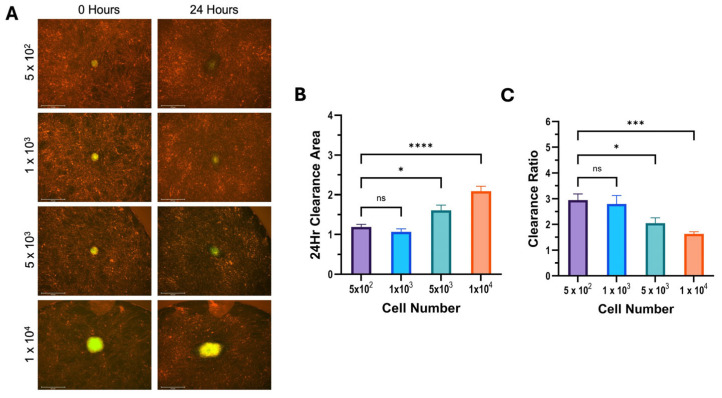
Increasing hEM3 cell number increases cleared area but decreases the clearance ratio. A mesothelial clearance assay was performed, plating hEM3 cells at the indicated spheroid cell numbers. (**A**) Representative images of 0 and 24 h of mesothelial clearance using the 4× objective. (**B**) Clearance area and (**C**) clearance ratio were determined. Data represent a minimum of 5 spheroids per assay, N = 3; ns > 0.05, * *p* ≤ 0.05, *** *p* ≤ 0.001, **** *p* ≤ 0.0001.

**Figure 6:**
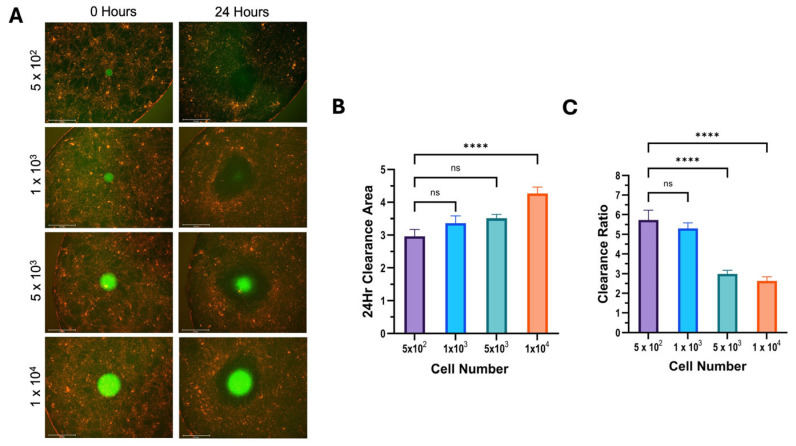
Increasing OVCAR8 cell number increases cleared area but decreases clearance ratio. A mesothelial clearance assay was performed, plating OVCAR8 cells at the indicated spheroid cell numbers. (**A**) Representative images of 0 and 24 h of mesothelial clearance using the 4× objective. (**B**) Clearance area and (**C**) clearance ratio were determined. Data represent a minimum of 5 spheroids per assay, N = 3; ns > 0.05, **** *p* ≤ 0.0001.

The results of the mesothelial clearance assay using differing cell numbers were consistent across all cell lines used. We found that increasing the number of cells in a spheroid increased the cleared area in 12Z cells (Figure 4), hEM3 cells (Figure 5), and OVCAR8 cells (Figure 6). Most notably, the results demonstrated that larger spheroids plated with a higher cell count had the smallest clearance ratio, whereas spheroids with lower cell counts had the highest clearance ratio. These results suggest that the extent to which a spheroid can perform mesothelial clearance is dependent on its initial size and the number of cells it is made up of. Based on the NucBlue and PI staining of all spheroids (Figure 2), the larger spheroids appear to have more dead cells than smaller ones, which could account for the inverse relationship between initial spheroid size and clearance ratio.

### 3.4. Analysis of Potential Pharmacological Inhibitors of Mesothelial Clearance

**Figure 7:**
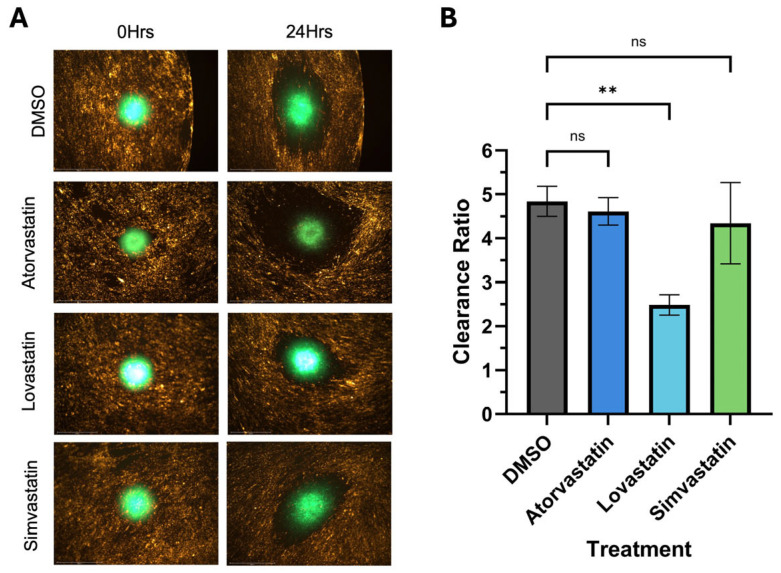
Treating 12Z spheroids with lovastatin reduces the amount of mesothelial clearance. 12Z cells were treated with 10 µM of either atorvastatin, lovastatin, or simvastatin for 24 h prior to starting the clearance assay. DMSO was used as a vehicle control. 12Z cells were allowed to clear for 24 h. Mesothelial clearance was imaged using the 4× objective (**A**), and clearance ratio was determined (**B**). Data represent a minimum of 8 spheroids per N, N = 3; ns > 0.05, ** *p* ≤ 0.01.

**Figure 8:**
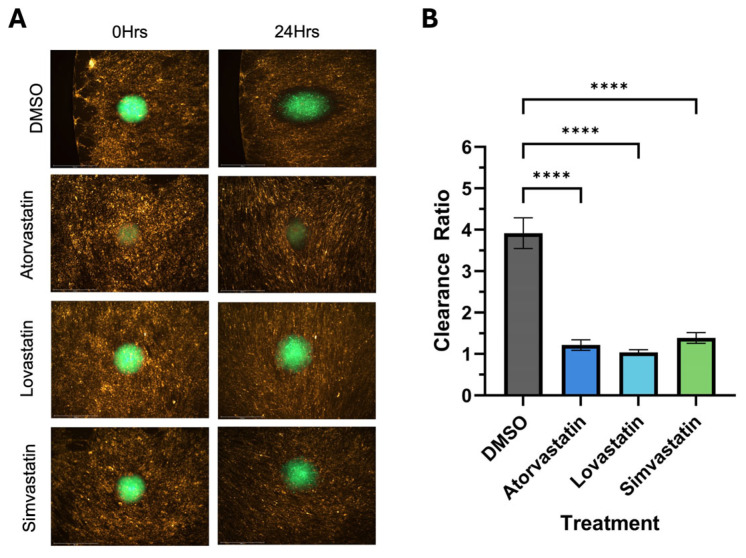
Treating hEM3 spheroids with statins significantly reduces the ability of the spheroids to undergo mesothelial clearance. hEM3 cells were treated with 10 µM of either atorvastatin, lovastatin, or simvastatin for 24 h prior to starting the clearance assay. DMSO was used as a control. hEM3 cells were allowed to clear for 24 h. Mesothelial clearance was imaged using the 4× objective (**A**), and clearance ratio was determined (**B**). Data represent a minimum of 8 spheroids per assay, N = 3; **** *p* ≤ 0.0001.

Many 2D cell culture models fail to accurately represent the complexity of the physical environment surrounding tissues in the body. This lack of sophistication is believed to be a main contributing factor to the high failure rate in drug discovery. We wanted to ensure our in vitro mesothelial clearance assay would be able to assess the ability of potential clearance inhibitors for endometriosis lesions. Statins have been suggested as a possible treatment for endometriosis [25]; therefore, we wanted to determine the effects of statin treatment on spheroids in our mesothelial clearance assay. To accomplish this, we treated 12Z and hEM3 spheroids with 10 mM of either atorvastatin, lovastatin, or simvastatin for 24 h prior to adding them on top of the LP9 mesothelial cell monolayer. The results for the 12Z spheroids demonstrated a significant reduction in clearance ratio when treated with lovastatin but no significant reduction in clearance ratio when treated with atorvastatin or simvastatin (Figure 7). In contrast, hEM3 spheroids showed a significant decrease in clearance ratio when treated with all three statins (Figure 8).

## 4. Discussion

Endometriosis is a painful gynecological disease whose pathology is poorly understood. Endometriotic lesions bypass the peritoneal mesothelial cell layer in order to facilitate their attachment to organs within the female reproductive system [26]. While most endometriotic lesions are found within the pelvic area, there have been some documented cases of extra-abdominal lesions [1]. The poorly understood process of endometriosis development presents an important area in the endometriosis field that has yet to be fully investigated. Previous studies have generated 3D spheroids made up of various combinations of endometriotic and normal endometrial stromal cells [13]. In these studies, researchers have demonstrated the ability of spheroids, either made up of normal endometrial stromal cells alone or in combination with endometriotic cells, to attach and invade past a monolayer of mesothelial cells [13]. What these studies did not investigate was the direct interaction of endometriotic cells with the mesothelial cell layer. In addition, the mechanisms responsible for the attachment of endometriotic lesions to the peritoneal mesothelial cell layer have yet to be uncovered.

We have developed an adaptable mesothelial clearance assay that can be used to model the interaction of spheroids with a monolayer of mesothelial cells. First, we cultured 12Z endometriotic epithelial cells and hEM3 epithelial cells in 3D cell culture using low-adhesion U-bottom plates. Both cell lines formed round spheroids with smooth outer borders, similar to OVCAR8 ovarian cancer cells, which we used as a reference since we previously determined they form spheroids. After we determined that our 12Z and hEM3 cells could form spheroids, we tested their ability to clear a monolayer of LP9 mesothelial cells and found that they were indeed able to disrupt the LP9 cell monolayer and push apart the mesothelial cells. The spheroids created a gap in the monolayer, which the cells that made up the spheroids started to disperse into. The results from this study using our mesothelial clearance assay have provided evidence to support our theory that endometriosis cell spheroids can undergo mesothelial clearance.

To ensure the clearance that occurred was not due to a co-culture effect, we tested the ability of Ishikawa endometrial cancer cells and ZR-75 breast cancer cells to clear the LP9 monolayer. Ishikawa spheroids were unable to disperse into the monolayer and only cleared the area beneath the spheroid. Notably, the Ishikawa cells formed irregular spheroids, which may have influenced their clearance capability. We also examined the breast cancer cell line ZR-75, which makes uniform spheroids, and these were able to create a gap in the monolayer and appeared to disperse slightly beyond the spheroid boundary (Supplemental Figure S5). This may not be surprising as a small number of breast cancer patients have been found to have metastases in the peritoneal cavity [27]. To further assess the potential co-culture effects, we created a monolayer of mouse embryonic fibroblasts and assessed the ability of the 12Z and hEM3 cells to clear these cells (Supplemental Figure S6). In all cases, the spheroids were unable to clear beyond the spheroid boundary. They remained tightly packed and did not disperse outward like the spheroid cells did with mesothelial cells. This suggests that the clearance by the endometrial spheroids is not due solely to a co-culture effect. Moreover, a study conducted by Davidowitz et al. found that not all ovarian cancer cell spheroids could clear a mesothelial cell monolayer [28]. The ability of ovarian cancer spheroids to clear the mesothelial layer was correlated with the expression of genes relating to the epithelial-to-mesenchymal transition (EMT) [28]. It would be interesting to further investigate the expression of EMT-related genes in the 12Z and hEM3 spheroids during clearance to unveil any similarities they have with the ovarian cancer spheroids and differences between the 12Z and hEM3 cell lines.

Interestingly, our data determined that the ratio of clearance area compared to the initial spheroid area had an inverse relationship to the cell seeding density and, therefore, the size of the original spheroid. Lastly, we tested the clearance ability of 12Z and hEM3 spheroids after treatment with three different statins. This demonstrated that our clearance assay could be used as a model for different drug treatments on endometriosis lesions. Our assay provides a straightforward approach that can be modified to investigate the role of different molecular pathways involved in the progression of endometriosis.

Previously, Witz et al. compared the ability of antegrade to shed menstrual endometrium from women without endometriosis and demonstrated that both endometrial stromal and epithelial cells were able to attach to human peritoneum explants [29]. Kavoussi et al. used the endometrial epithelioid cell line EM42 and reported that attachment to a monolayer of LP9 mesothelial cells occurred [30]. These studies demonstrated that epithelial cells could attach to mesothelial cells, but again, they did not use endometriosis cells. Song et al. published a study where they used endometriotic epithelial cells (12Zs) and endometriotic stromal cells to form spheroids [13]. These cells self-organized into spheroids that had a stromal cell core that was surrounded by multiple layers of epithelial cells after one week of growing [13]. Interestingly, using a Matrigel/LP9 cell layer to mimic invasion, the endometriotic stromal cells were the driving force in the disruption of the mesothelial cell layer even though the 12Z epithelial cells were located on the outermost layers of the spheroid [13]. However, they did not measure the ability of the endometriotic epithelial cells to interact with the LP9 cell layer on their own. We determined that when transferred on top of a mesothelial cell monolayer, both 12Z and hEM3 cell spheroids can push away the mesothelial cells, creating a space that they can then colonize. These results reveal an important element in the development of endometriosis that augments what other studies have previously published.

Endometriosis is a complex disease whose presence is due to an unknown combination of multiple factors [7]. Endometriosis lesion formation and heterogeneity can differ greatly from case to case [1,7,11]. It is therefore possible that epithelial cells from normal and endometriotic endometrium may have an intrinsic ability to clear the mesothelial cell layer that lines the peritoneal cavity. In different conditions, different cell types could potentially drive mesothelial clearance, with some cell types having a stronger ability than others. In our clearance assays, 12Z cells made consistently larger spheroids compared to hEM3 cells. In addition, the area cleared by the 12Z spheroids was also larger than the area cleared by hEM3 spheroids. This resulted in an overall larger clearance ratio value for 12Z spheroids than hEM3 spheroids. Overall, our results support our hypothesis that 12Z and hEM3 epithelial cells can form spheroids in 3D culture that are capable of performing mesothelial clearance. The differences in clearance ratio values between these two cell lines could potentially be due to one cell line being endometriotic cells and the other being normal cells. Regardless, we have demonstrated that endometrial epithelial cells can perform mesothelial clearance, which can help us better understand how endometriosis lesions occur not only within the female reproductive system but around the peritoneal cavity and may suggest that mesothelial clearance is needed prior to moving to extra-pelvic locations.

Solid tumors have unique structures and arrangements of cells that result in multiple layers of cells [31]. The external layer of cells has more access to oxygen and nutrients within the tumor microenvironment, which causes them to proliferate more rapidly [31]. In contrast, the middle layer of cells has less access to oxygen and nutrients and is in a senescent state [31]. The innermost layer of solid tumors often has no access to oxygen or nutrients, causing them to become necrotic [31]. To assess the composition of the 12Z and hEM3 cell spheroids, we stained them with NucBlue and propidium iodine. As expected, the OVCAR8 cell spheroids had an outer layer of living cells, while the cells making up the inner layer of the spheroid were dead. This pattern was also observed in the 12Z cell spheroids. Interestingly, the spheroids formed from hEM3 cells showed no definitive separation of dead and living cells within the inner and outer layers of the spheroid. This could potentially explain why hEM3 cells formed smaller spheroids and had an overall smaller clearance ratio when compared to 12Z cell spheroids. The difference between these cell lines is that 12Z cells are endometriotic cells, whereas hEM3 cells are not, which could explain the differences we see in the 3D spheroid formation. To explore why these differences in spheroid composition occur, future research efforts should focus on 3D spheroid gene expression.

Three-dimensional spheroids have become popular in vitro models for drug testing in cancer fields due to the similarities they show with not only the morphology of solid tumor spheroids but gene expression and drug response as well [31]. After determining that endometriotic spheroids could undergo mesothelial clearance, we wanted to determine if our assay could be used in combination with drug treatments to demonstrate the effectiveness of the drugs on inhibiting mesothelial clearance. Statins, a class of medication typically prescribed to help lower cholesterol, are also being tested as a potential treatment for cancer in clinical trials [32]. The effectiveness of statins for cancer treatment seems to vary by statin type and cancer type [32]. Due to their antioxidant characteristics, statins have also been investigated as a potential treatment for endometriosis, as it is a highly inflammatory disease with increased levels of oxidative stress [1,33]. Using a rat model of endometriosis, Oktem et al. reported that a high dose of atorvastatin lowered the mean area of lesion size compared to the control group [33]. Another statin, lovastatin, was found to inhibit the proliferation and angiogenesis of endometrial stromal cells in a dose-dependent manner [25]. In another study using nude mice, simvastatin also decreased the number and size of endometriotic lesions in a dose-dependent manner [34]. Acknowledging that these statins were administered after endometriosis was induced in the mouse and rat models and after endometriotic tissues from patients were collected and cultured in vitro, these results may speak more to the treatment of pre-established endometriosis lesions rather than as a method of endometriosis prevention.

In a literature review by Gibran et al., several studies highlighted simvastatin treatment on endometriotic stromal cells, resulting in a decrease in proliferation and increases in apoptosis, cell shrinkage, and the breakdown of the cytoskeleton [35,36,37]. These data suggest that statins could be a useful treatment for endometriosis. Therefore, we treated 12Z and hEM3 spheroids with atorvastatin, lovastatin, and simvastatin to ascertain if they had any impact on the clearance ability of these cell lines. All statins significantly decreased the clearance ratio of hEM3 spheroids, but only lovastatin significantly reduced the clearance ratio of 12Z spheroids. This outcome may reflect the differences between the cell lines, 12Z cells being endometriosis cells and hEM3 cells being normal endometrium cells, or it may reflect differences between the statins themselves. While some data suggest that statins may be beneficial for endometriosis, there is some evidence that statin use is associated with endometriosis. Jiao et al. investigated ovary- and uterus-related adverse events associated with statin use based on available data from the FDA Adverse Event Reporting System (FAERS) [38]. This analysis took into consideration not only statins as a class but also seven individual statins and their reported adverse events. For example, statins as a class were reported to reduce the proliferation, viability, and migration of endometrial cells [38]. Individually, atorvastatin and simvastatin were reported to be associated with an increased risk of endometriosis, while lovastatin use was not [38]. This could explain why only lovastatin significantly reduced the mesothelial clearance done by our 12Z spheroids when all three statins significantly reduced the amount of mesothelial clearance the hEM3 spheroids did. It is also interesting to note that compared to atorvastatin and simvastatin, Jiao et al. found that lovastatin did not result in as many adverse events overall [38]. Therefore, more research is needed to understand the benefits and risks of statins and endometriosis. Regardless, we have shown that the mesothelial clearance model we developed has a promising use in testing possible pharmacological therapies for endometriosis prior to clinical trials.

There are currently no definitive biomarkers for endometriosis [1]. Endometriosis is an inflammatory disease, which has led researchers to study altered levels of pro-inflammatory cytokines in endometriosis [1,39]. Cytokines can influence cell proliferation and differentiation and are important for the progression of endometriosis [40]. While multiple studies have found altered cytokine levels in patients with endometriosis, these results are not consistent across multiple studies and the menstrual phases of patients [2]. Instead of focusing solely on the altered levels of cytokines, investigating related pathways and key proteins involved with the cytokines of interest could shed light on endometriotic lesion formation and attachment. Our mesothelial clearance model enables researchers to accomplish this. For example, increased levels of the cytokine interleukin-6 (IL-6) have been reported in the serum of patients with endometriosis, leading to persistent activation of the signal transducer and activator of transcription 3 (STAT3) protein [40,41]. Our assay could be used to assess IL-6-activated STAT3 importance in mesothelial clearance in two ways. First, by stimulating the spheroid cells with IL-6 before transferring them onto the LP9 mesothelial layer and comparing the amount of clearance between stimulated and unstimulated spheroids, and second, through knocking down *STAT3* in the spheroids for the mesothelial clearance assay. This will allow researchers to determine if STAT3 activated via IL-6 is required for mesothelial clearance or not. This will help to gain a deeper understanding of how endometriosis lesions are able to attach to and clear the mesothelial cell layer in the peritoneal cavity, resulting in lesions on organs within the peritoneal cavity and in extra-pelvic regions as well. Lastly, as mesothelial clearance and invasion are two separate steps, our method can be used to separate the genes specific to mesothelial clearance versus invasion.

## 5. Conclusions

Overall, the data presented here demonstrate that endometriotic and normal endometrial epithelial cells are capable of undergoing mesothelial clearance. Importantly, spheroids made up of fewer numbers of cells had a smaller clearance area but a larger clearance ratio, whereas spheroids made up of a greater number of cells had a larger clearance area but a smaller clearance ratio. This highlights the importance of comparing the clearance ratio instead of just analyzing the cleared area. Here, we have shown that endometriotic epithelial cells can also clear a mesothelial cell monolayer. This is important for endometriotic lesions as they are found attached to organs within the peritoneal cavity. Some cases of extra-pelvic endometriotic lesions have been documented; therefore, further studies should be conducted to determine if endometriotic lesions can break through the extracellular matrix below, as ovarian cancer cells can. This will shed light on the elusive pathology of endometriosis and how lesions spread and better inform research aimed at treating this disease.

## Data Availability

Data are available at https://figshare.com/articles/figure/Complete_Image_Sets/28796033.

## Supplementary Materials

The following supporting information can be downloaded at https://www.mdpi.com/article/10.3390/cells14100742/s1. Figure S1: Phase-contrast images of cells in 2D and 3D. Figures S2–S4: Time-lapse images of 12Z, hEM3, and OVCAR8 cells, respectively, undergoing mesothelial clearance. Figure S5: Mesothelial clearance by Ishikawa and ZR-75 cells. Figure S6: Clearance of MEFs by 12Z, hEM3, and OVCAR8 spheroids.

## Abbreviations

The following abbreviations are used in this manuscript:

3D	Three-dimensional
2D	Two-dimensional
ULA	Ultra-low attachment
PI	Propidium iodide
DMSO	Dimethyl sulfoxide
