# Supplementary material for: Endometriosis Cell Spheroids Undergo Mesothelial Clearance in a Similar Manner to Ovarian Cancer Cell Spheroids
Source: Cells. 2025 May 19;14(10):742. doi: 10.3390/cells14100742 (PMC12110144; doi:10.3390/cells14100742)
Supplement: Supplementary file 1 [file cells-14-00742-s001.zip › cells-3615757-supplementary.pptx]

## Slide 1
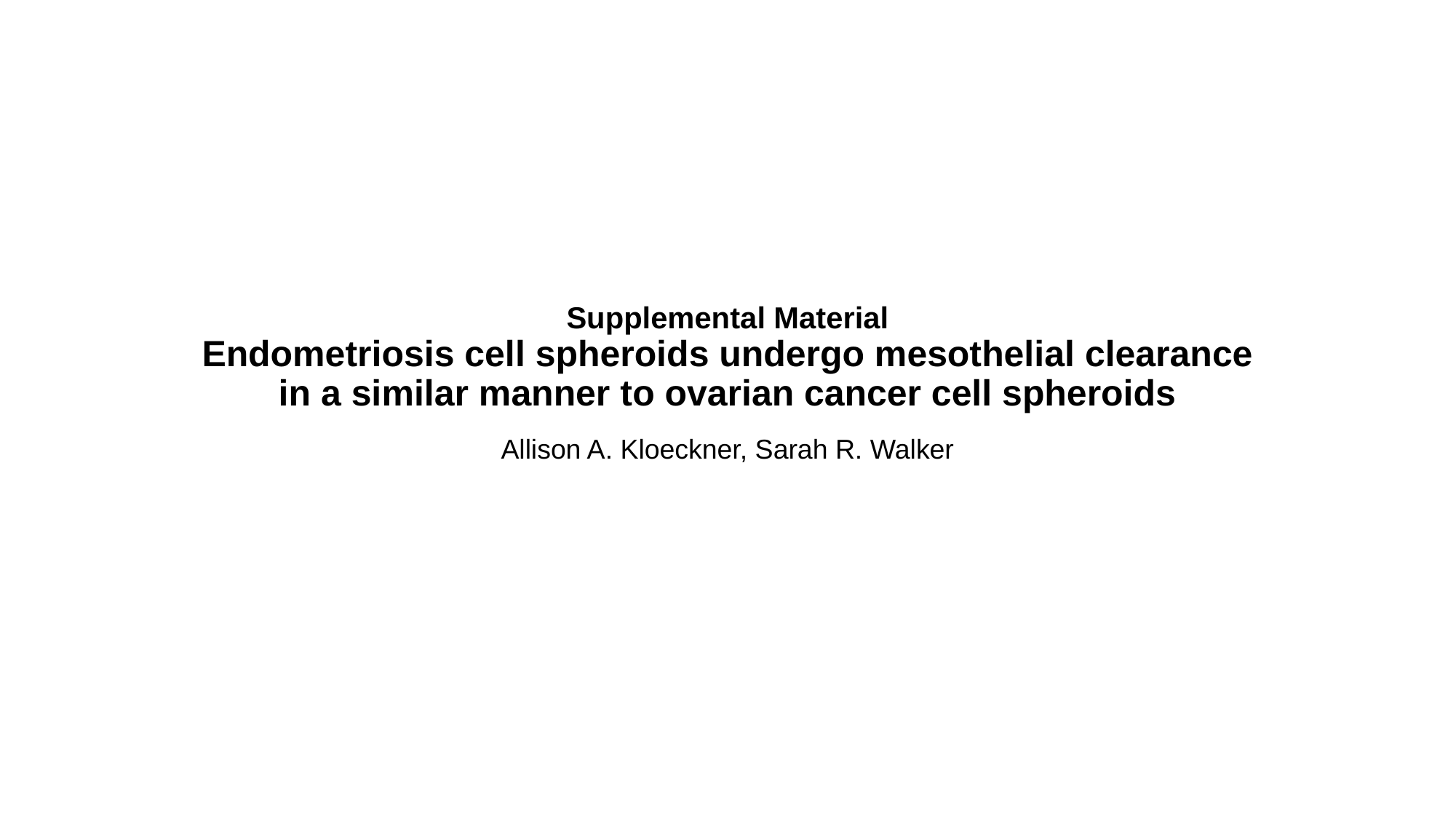

# Supplemental Material Endometriosis cell spheroids undergo mesothelial clearance in a similar manner to ovarian cancer cell spheroids
Allison A. Kloeckner, Sarah R. Walker

## Slide 2
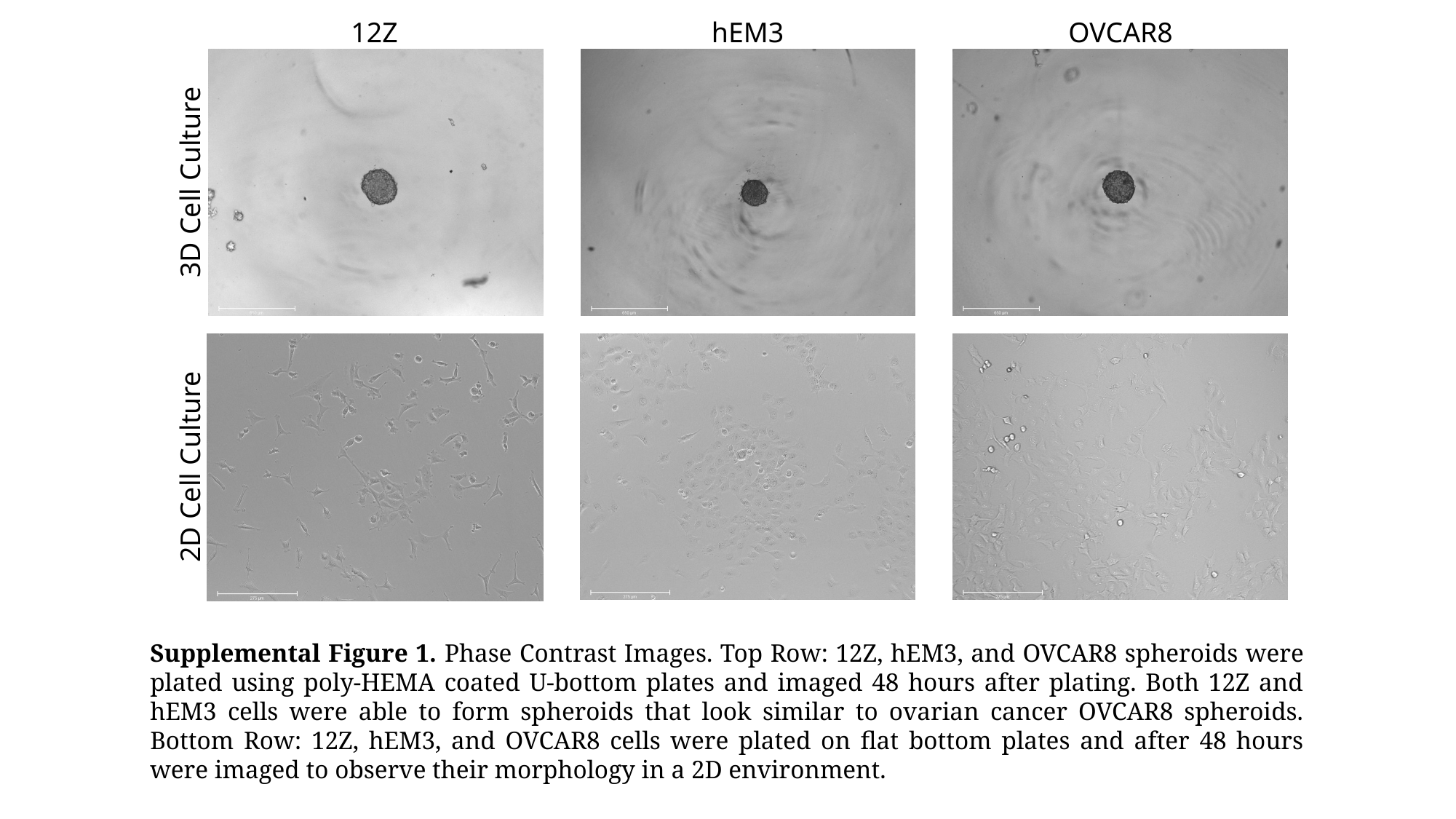

12Z
hEM3
OVCAR8
3D Cell Culture
2D Cell Culture
Supplemental Figure 1. Phase Contrast Images. Top Row: 12Z, hEM3, and OVCAR8 spheroids were plated using poly-HEMA coated U-bottom plates and imaged 48 hours after plating. Both 12Z and hEM3 cells were able to form spheroids that look similar to ovarian cancer OVCAR8 spheroids. Bottom Row: 12Z, hEM3, and OVCAR8 cells were plated on flat bottom plates and after 48 hours were imaged to observe their morphology in a 2D environment.

## Slide 3
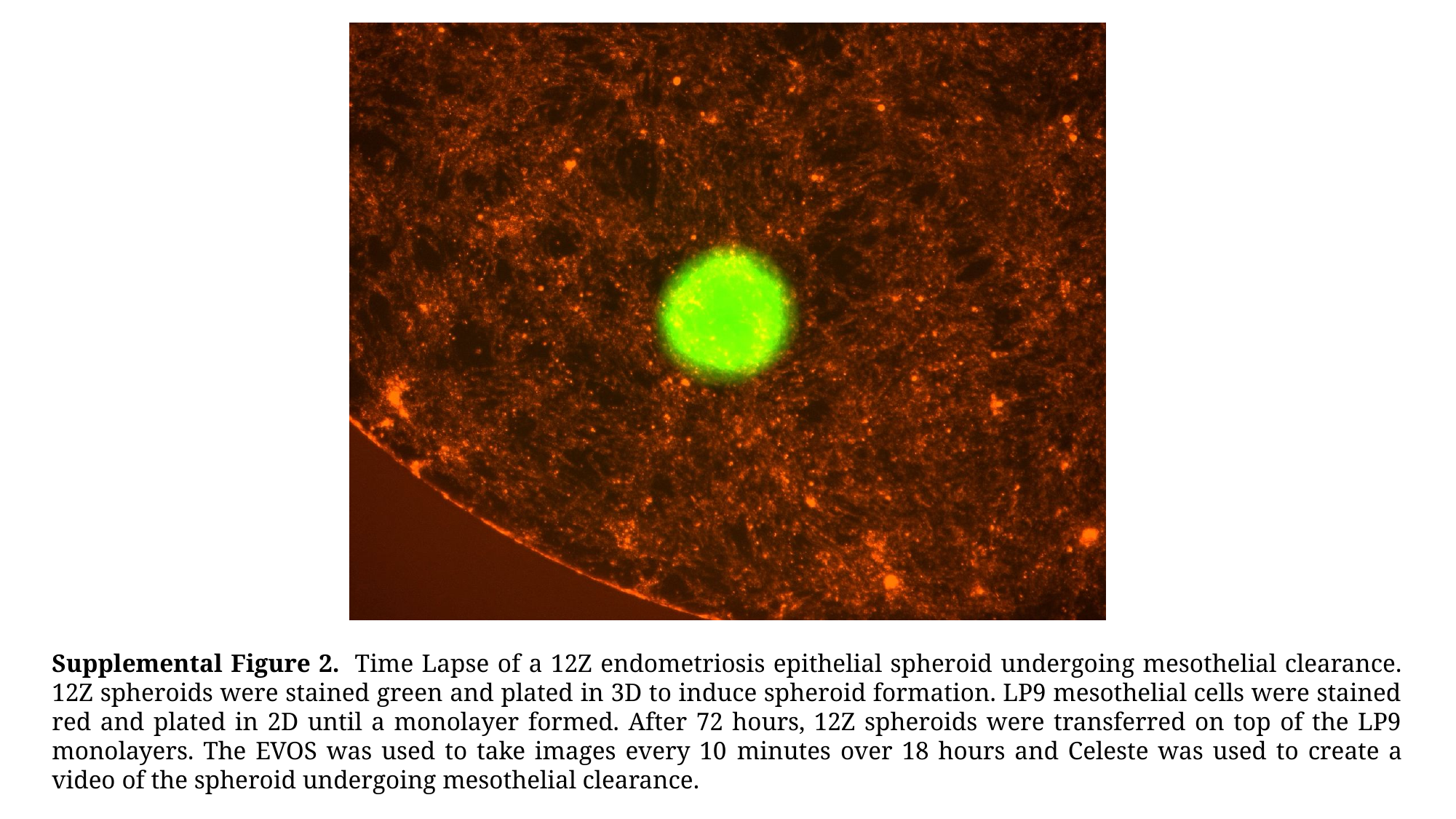

Supplemental Figure 2.  Time Lapse of a 12Z endometriosis epithelial spheroid undergoing mesothelial clearance. 12Z spheroids were stained green and plated in 3D to induce spheroid formation. LP9 mesothelial cells were stained red and plated in 2D until a monolayer formed. After 72 hours, 12Z spheroids were transferred on top of the LP9 monolayers. The EVOS was used to take images every 10 minutes over 18 hours and Celeste was used to create a video of the spheroid undergoing mesothelial clearance.

## Slide 4
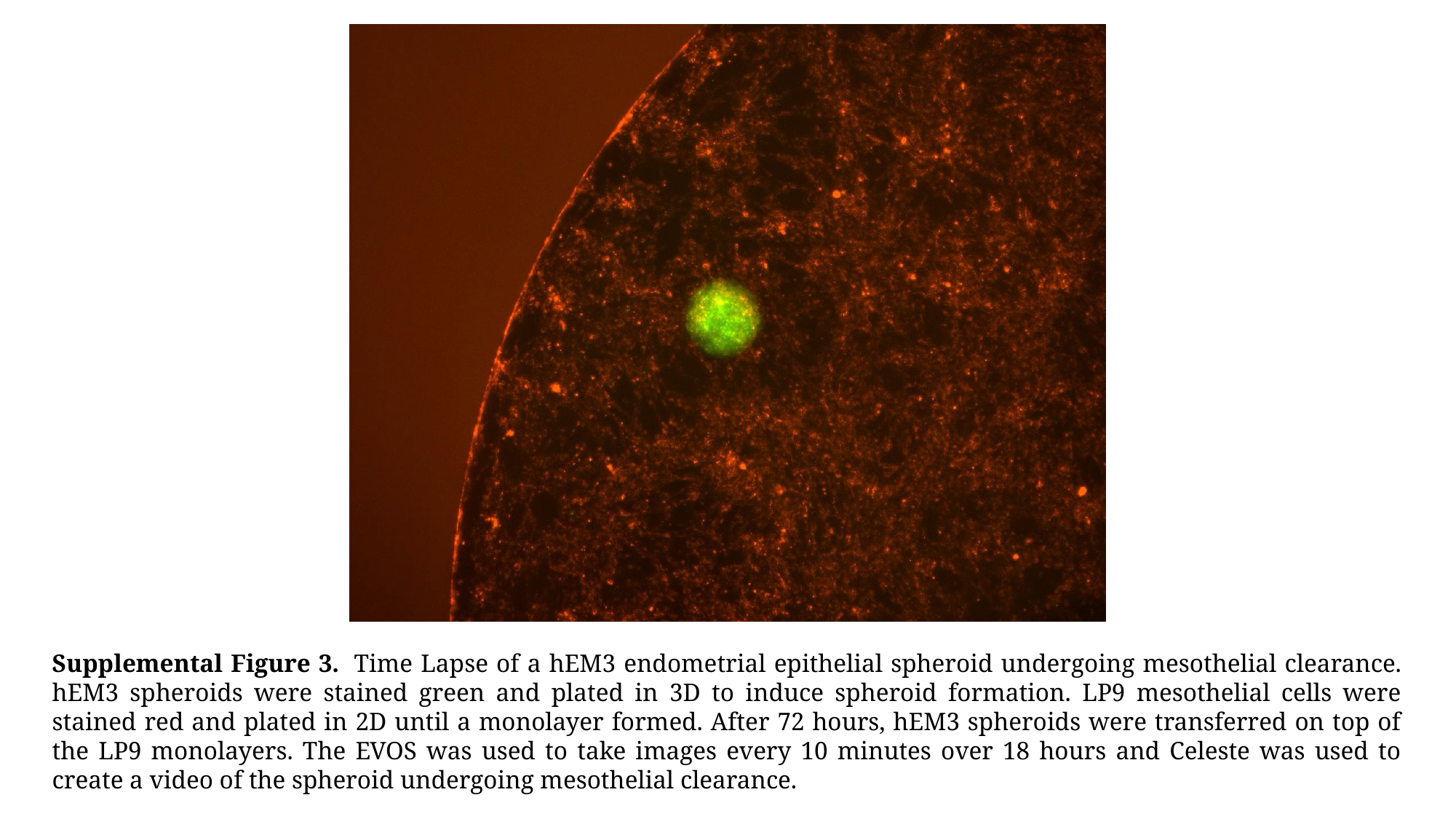

Supplemental Figure 3.  Time Lapse of a hEM3 endometrial epithelial spheroid undergoing mesothelial clearance. hEM3 spheroids were stained green and plated in 3D to induce spheroid formation. LP9 mesothelial cells were stained red and plated in 2D until a monolayer formed. After 72 hours, hEM3 spheroids were transferred on top of the LP9 monolayers. The EVOS was used to take images every 10 minutes over 18 hours and Celeste was used to create a video of the spheroid undergoing mesothelial clearance.

## Slide 5
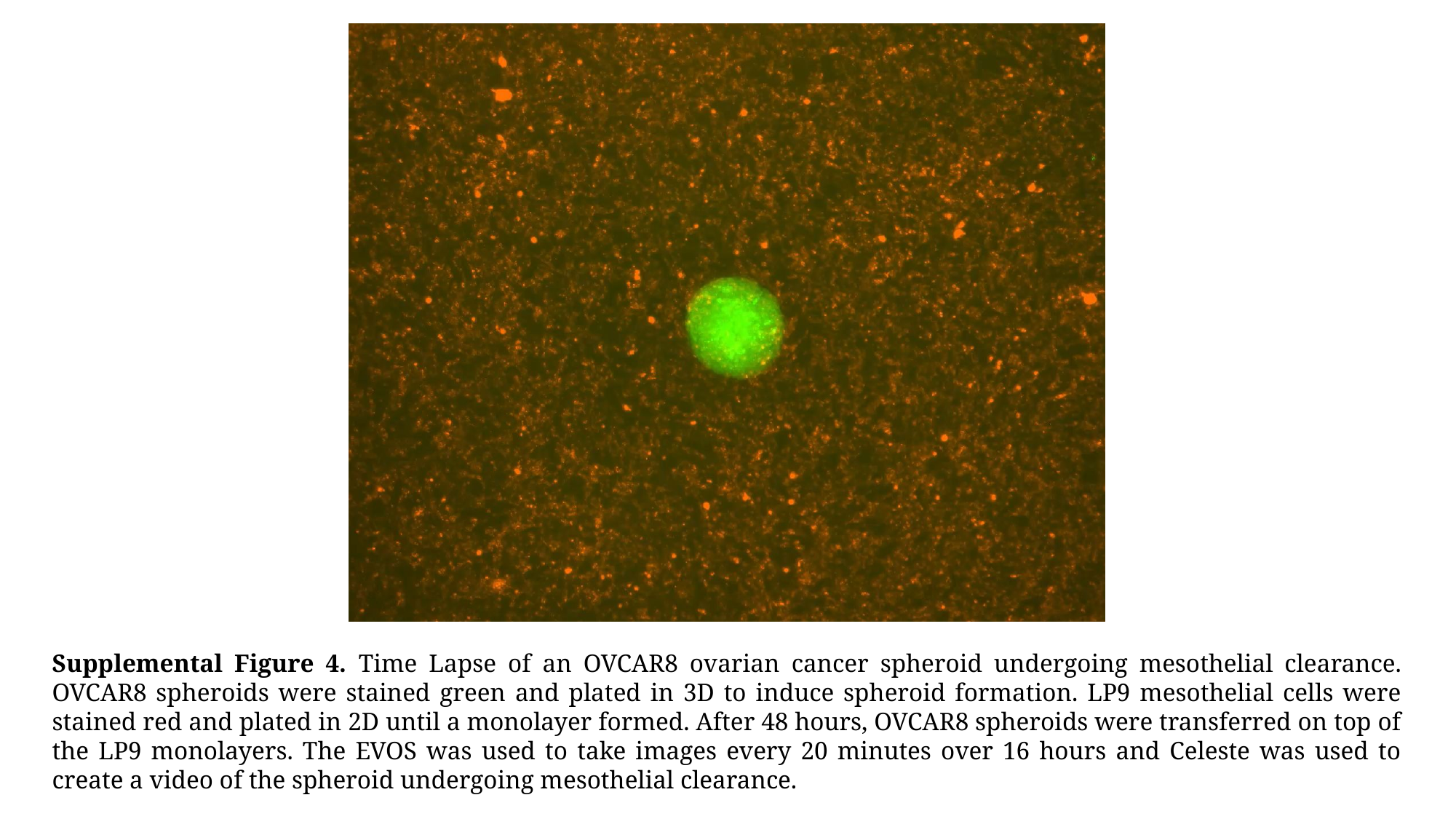

Supplemental Figure 4. Time Lapse of an OVCAR8 ovarian cancer spheroid undergoing mesothelial clearance. OVCAR8 spheroids were stained green and plated in 3D to induce spheroid formation. LP9 mesothelial cells were stained red and plated in 2D until a monolayer formed. After 48 hours, OVCAR8 spheroids were transferred on top of the LP9 monolayers. The EVOS was used to take images every 20 minutes over 16 hours and Celeste was used to create a video of the spheroid undergoing mesothelial clearance.

## Slide 6
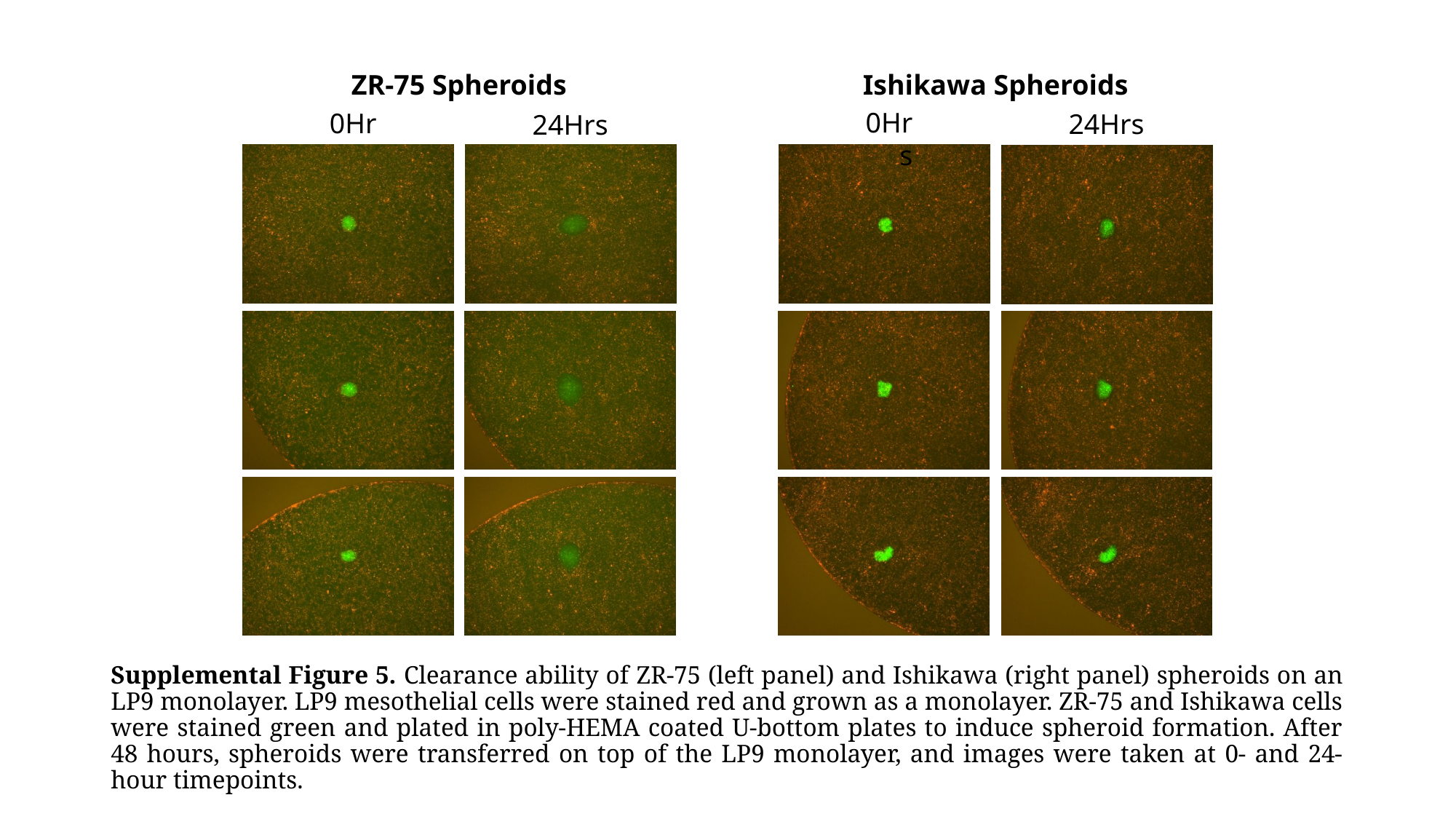

ZR-75 Spheroids
0Hrs
24Hrs
Ishikawa Spheroids
0Hrs
24Hrs
# Supplemental Figure 5. Clearance ability of ZR-75 (left panel) and Ishikawa (right panel) spheroids on an LP9 monolayer. LP9 mesothelial cells were stained red and grown as a monolayer. ZR-75 and Ishikawa cells were stained green and plated in poly-HEMA coated U-bottom plates to induce spheroid formation. After 48 hours, spheroids were transferred on top of the LP9 monolayer, and images were taken at 0- and 24-hour timepoints.

## Slide 7
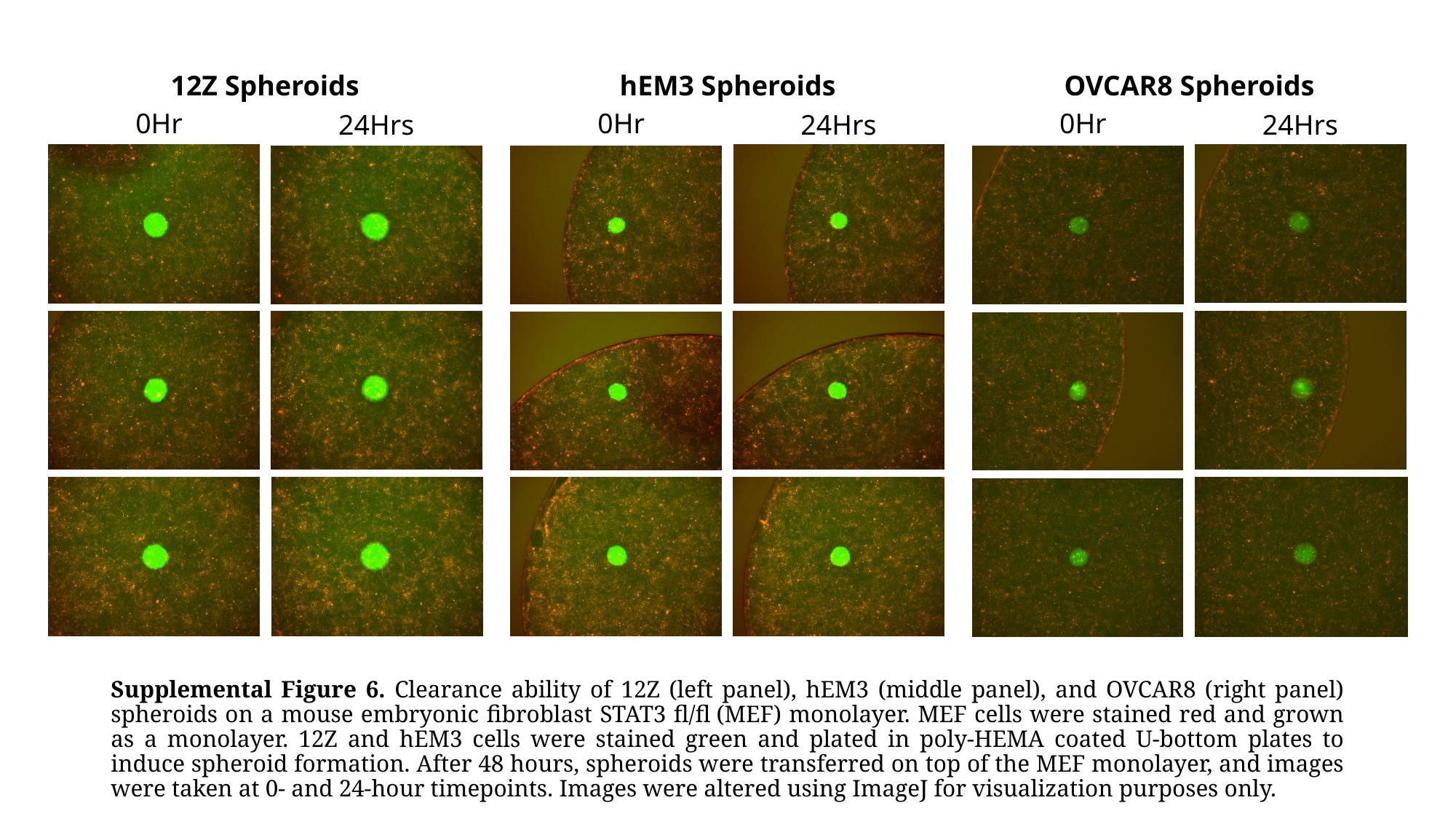

12Z Spheroids
0Hrs
24Hrs
hEM3 Spheroids
0Hrs
24Hrs
OVCAR8 Spheroids
0Hrs
24Hrs
# Supplemental Figure 6. Clearance ability of 12Z (left panel), hEM3 (middle panel), and OVCAR8 (right panel) spheroids on a mouse embryonic fibroblast STAT3 fl/fl (MEF) monolayer. MEF cells were stained red and grown as a monolayer. 12Z and hEM3 cells were stained green and plated in poly-HEMA coated U-bottom plates to induce spheroid formation. After 48 hours, spheroids were transferred on top of the MEF monolayer, and images were taken at 0- and 24-hour timepoints. Images were altered using ImageJ for visualization purposes only.
